# Radiant Heat in Ulcers of the Leg

**Published:** 1897-09

**Authors:** 


### Radiant Heat in Ulcers of the Leg.

Dr. Colleville, professor in the Rheims School of Medicine, treats ulcer of the leg by exposure to heat without any very elaborate apparatus. All that is required is a square plate of metal which will stand heating, and a Bunsen burner. The blue flame of the latter impinges on the metal so as to bring it to a dull red heat, and the ulcer is exposed to the action of this at a distance of about ten inches, the rest of the limb being protected by bandages. The temperature is about 45 degrees C., which is easily borne, and the flame is regulated so as to maintain this temperature at the wound during the whole of the sitting, which lasts for from twenty minutes to an hour. At the conclusion the surface is found to be glaced over, large granulations being visible through the semitransparent coating. It is best to leave the ulcer exposed to the air for some time, and when it is dressed care should be taken that its surface is not touched by the aseptic gauze or other material used. Some improvement is generally experienced by the patient even after the first sitting, and cicatrization is completed in from five to twenty-five applications. In the latter sittings, when the ulcer is nearly healed, a more moderate degree of heat may be emptoyed. Where gas is not available the heat of the sun or that of a fire may be utilized. Dr. Colleville attributes the beneficial effect of this method to the combined action of heat, light and ventilation.—*Journal Am. Med. Asso.*

